# Current Trends in the Application of Nanomaterials for the Removal of Emerging Micropollutants and Pathogens from Water

**DOI:** 10.3390/molecules25092016

**Published:** 2020-04-26

**Authors:** Petros Kokkinos, Dionissios Mantzavinos, Danae Venieri

**Affiliations:** 1 Department of Chemical Engineering, University of Patras, Caratheodory 1, University Campus, GR-26504 Patras, Greece; 2 School of Environmental Engineering, Technical University of Crete, GR-73100 Chania, Greece

**Keywords:** nanomaterials, catalytic processes, degradation, disinfection, carbon based, graphene, metal oxides, composites, metal organic frameworks, pharmaceuticals, microorganisms

## Abstract

Water resources contamination has a worldwide impact and is a cause of global concern. The need for provision of clean water is becoming more and more demanding. Nanotechnology may support effective strategies for the treatment, use and reuse of water and the development of next-generation water supply systems. The excellent properties and effectiveness of nanomaterials make them particularly suitable for water/wastewater treatment. This review provides a comprehensive overview of the main categories of nanomaterials used in catalytic processes (carbon nanotubes/graphitic carbon nitride (CNT/g-C_3_N_4_) composites/graphene-based composites, metal oxides and composites, metal–organic framework and commercially available nanomaterials). These materials have found application in the removal of different categories of pollutants, including pharmaceutically active compounds, personal care products, organic micropollutants, as well as for the disinfection of bacterial, viral and protozoa microbial targets, in water and wastewater matrices. Apart from reviewing the characteristics and efficacy of the aforementioned nanoengineered materials for the removal of different pollutants, we have also recorded performance limitations issues (e.g., toxicity, operating conditions and reuse) for their practical application in water and wastewater treatment on large scale. Research efforts and continuous production are expected to support the development of eco-friendly, economic and efficient nanomaterials for real life applications in the near future.

## 1. Introduction

**Figure 1:**
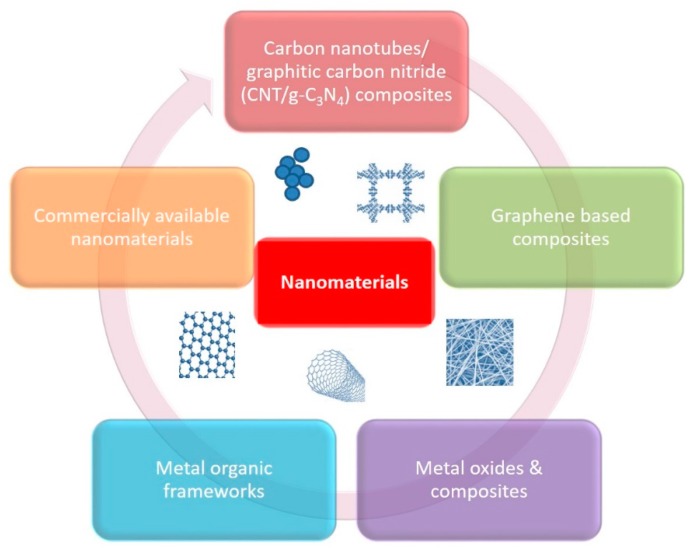
Different categories of nanomaterials presented in the present review (carbon nanotubes/graphitic carbon nitride (CNT/g-C_3_N_4_) composites/graphene-based composites, metal oxides and composites, metal organic frameworks and commercially available nanomaterials).

New strategies of water and wastewater treatment with the use of nanosized materials have been explored during the last few decades. Nanotechnology supports the development of next-generation water supply systems. Nanoengineered materials can adsorb and/or degrade various pollutants (metal ions, dyes, pesticides, pharmaceuticals, other organic pollutants, etc.) and possess interesting antimicrobial properties against waterborne microbes (bacteria, viruses, protozoa, etc.). Extraordinary features of nanomaterials supporting their potential for efficient hydric resources remediation include high surface area, excellent mechanical properties, lower cost and energy requirements, greater chemical reactivity, recyclability, ease of fabrication, and functionalization [1,2,3,4,5]. Das et al. reviewed the trends in nanomaterials applications in environmental monitoring and remediation and underlined the efficiency of these nanotools, along with the need to avoid further environmental contamination due to their use [6]. Similarly, Karn et al. reported on the benefits and potential risks of nanomaterials and claimed that in the absence of definitive risk data, nanotechnology should be generally viewed as more beneficial than harmful. Many different nanoscale materials have found environmental applications, such as nanoscale zeolites, metal oxides and titanium dioxide, carbon nanotubes and fibers, enzymes and various noble metals [7]. All these nanomaterials could be categorized as shown in Figure 1 (carbon nanotubes/graphitic carbon nitride (CNT/g-C_3_N_4_) composites/graphene-based composites, metal oxides and composites, metal organic frameworks and commercially available nanomaterials).

**Figure 2:**
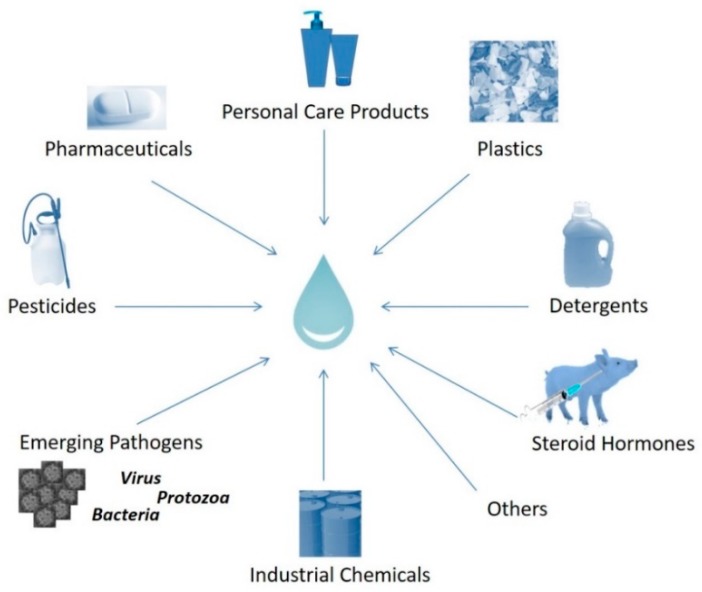
Water contaminants of emerging concern.

Wastewater is known to contain a wide range of pollutants (pharmaceuticals, surfactants, inhibitors, etc.), whose origin and properties can differ significantly (Figure 2). 

These emerging contaminants are continuously released into the environment as a result of their use in industry, agriculture, medical care, consumer goods and household activity. Most of these micropollutants and their metabolites are poorly biodegradable and only insufficiently removed during wastewater treatment processes and, thus, they are detected in treated wastewater and aquatic environment, worldwide [8,9,10,11,12,13,14,15,16,17,18,19,20]. Apart from wastewater, other important sources of micropollutants include the runoff from agriculture and livestock areas, irrigation with treated wastewater, agricultural reuse of sewage sludge as fertilizer, the leakage from landfills, septic tanks and industrial waste systems [21]. Micropollutants are usually present in waters at trace levels (from ng/L to μg/L). Thus, their detection and quantification are a real challenge [8,21]. Ul-Islam et al. reviewed the advancements of magnetic nanoparticles in adsorption and degradation of organic pollutants [3]. The use of cellular extracts (from bacteria, fungi, algae and plants) as green reagents for the fabrication of nanomaterials has, currently, been recognized as a sustainable, energy efficient, ecologically friendly and low-priced approach for water and wastewater treatment. Gautam et al. reviewed recently the synthesis and applications of biogenic nanomaterials in such applications [1]. Similarly, Ali et al. reviewed the microbes-based fabricated biogenic nanoparticles (NPs) for water and wastewater treatment [22], while Hennebel et al. reported on the biogenic production of palladium nanoparticles [23].

Pharmaceuticals are a significant class of emerging environmental micropollutants [9,13,24,25]. Tons of pharmaceuticals are annually produced, consumed and released in the environment, and their presence in water bodies (surface water, groundwater, seawater and treated sewage effluents) is well-documented worldwide, consisting of an issue of great concern, because they negatively affect the growth, reproduction and behavior of non-target organisms and especially aquatic ones. Various ecotoxicological effects have been reported for pharmaceutical micropollutants [9,25]. Different compounds for human and veterinary use have been reported to be prescribed worldwide (5000 compounds for Europe and 10,000 for USA) [26]. The prevalence of more than 200 pharmaceutical compounds in river waters has been reported worldwide [9]. Fekadu et al. have recently reviewed the occurrence of pharmaceuticals in freshwater aquatic environments in Europe and Africa continents. Interestingly, numerous pharmaceuticals were detected at high concentration levels exceeding their ecotoxicity endpoints [26].

Any product with healthcare or medical purposes for humans and/or animals is broadly referred to as pharmaceutical and personal care product (PPCP) [27,28]. Due to their universal consumption, low human metabolic capability and improper disposal, along with the inability of wastewater treatment plants to effectively remove them, PPCPs are detected in water bodies at concentrations ranging between ng/L to μg/L. Despite their low concentrations, PPCPs may negatively affect human health and the environment [27]. Hopkins and Blaney reported on the need for improved wastewater treatment of antimicrobials, polycyclic musks and UV filters for the prevention of adverse effects on aquatic species [28].

Antibiotics are increasingly used in human and veterinary treatments; they are less metabolized and discharged through human and animal excretion into the wastewater and the soil. It is known that some antibiotics are only partially transformed in wastewater treatment plants and thus are continuously released into water bodies consisting a significant class of emerging water contaminants [12,27,29,30,31]. It has been reported that 63,151 tons of 250 different types of antibiotics are being used annually in human and veterinary medicines with ca 70% of them neither metabolized nor absorbed in human or animal body and thus excreted into the environment [9].

Organisms from the urban microbiome are continuously released to water recipients by sewer systems and urban runoff, and thus the imprint of the urban signature can be seen in contaminated waterways [32]. Authors underlined the need to prioritize the studies of urban aquatic microbiomes because of the resulting positive impact on both human and ecological health. Vittecoq et al. stressed out the need for the implementation of adequate water treatment and surveillance for the prevention of disease emergence in the Mediterranean basin [33]. During the last decades, at least 50 emerging infectious agents have been identified and approximately 10% of them are bacterial agents [34]. Water borne and non-fatal infections are responsible for approximately 200 million deaths annually [9]. Opportunistic pathogens including *Pseudomonas aeruginosa*, *Legionella pneumophila*, *Mycobacterium avium* and other non-tuberculous mycobacteria are responsible for an emerging waterborne disease problem with a significant annual economic cost. In an attempt to alert the drinking water community, Falkinham et al. reviewed the characteristics of opportunistic premise plumbing pathogens [35]. La Rosa et al. stressed out the potential of viruses to become emerging pathogens because of their biology (ability to infect new hosts and adjust to new environments). They focused on waterborne viruses representing potentially emerging agents [36]. Many different nanomaterials (e.g., nano-Ag, nano-TiO_2_, nano-ZnO, carbon nanotubes, etc.) possess significant antimicrobial characteristics, based on the generation of reactive oxygen species, the release of toxic metal ions and the damage of cell membrane integrity upon direct contact. These nanomaterials provide an effective alternative to the application of traditional disinfectants without the formation of harmful disinfection byproducts [37].

Although the toxicity and the linked negative effects of organic micropollutants (including solvents, polycyclic aromatic hydrocarbons (PAHs), bisphenol A, pesticides and organochlorine pesticides, alkyl phenols, polybromodiphenyl ethers and polychlorinated biphenyls) is well-documented, their production, use and spread is expected to be increased in the future. Interestingly, approximately 33 million organic and inorganic substances have been synthesized during the period 1907–2008, and about 4000 new compounds were being added to the list on daily basis. Approximately 4.6 million tons of different categories of pesticides (insecticides, fungicides, and herbicides, molluscicides, nematocides and rodenticides) are being sprayed annually, with a significant amount finding its way to water recipients [9].

The application of advanced treatment technologies for the upgrade of wastewater treatment plants, which aim to transform micropollutants into less harmful compounds or even to mineralize them, is a promising approach. Advanced oxidation processes (AOPs), such as photocatalysis, ozonation, sonolysis, electrochemical oxidation, Fenton and alike reactions, are based on the production of highly reactive oxygen species, and can be used as pre- or post-treatment to a biological process [10,12,14,21,25,27,30,38,39,40,41,42,43]. Kudnan and Chowdhure reported recently on the use of novel nanostructured photocatalysts for the environmental sustainability of wastewater treatments [44]. Zhao et al. reviewed the generation of sulfate radical using metal-free catalysts (carbon nanotubes, graphene, mesoporous carbon, activated carbon, activated carbon fiber and nanodiamond) [45]. Saqib et al. reviewed the enhancement of TiO_2_ photocatalysts through their modification with rare earth metals [46]. Solar photocatalytic ozonation has been reviewed by Beltrán et al. to highlight the significance of this hybrid process as a more sustainable water treatment technology for the abatement of emerging contaminants [47]. The practicability of concurrent abatement of pathogens and chemical pollutants by solar-enhanced AOPs has been reviewed by Tsydenova et al. [41]. Duan et al. presented the metal-free carbocatalysis in AOPs as a green remediation alternative to metal-based processes, characterized by poor stability and metal leaching [48]. Wols and Hofman-Caris reviewed the photochemical reaction constants for the degradation of organic micropollutants in water by photochemical AOPs [11].

## 2. Main Groups of Nanomaterials Used in Catalytic Processes

### 2.1. Carbon Nanotubes/ Graphitic Carbon Nitride (CNT/g-C_3_N_4_) Composites 

Carbon-based nanomaterials (such as carbon nanotubes graphene and graphitic carbon nitride) are excellent materials for environmental remediation applications [49]. They are characterized by eco-friendliness, earth-abundance, large surface area, high electrical conductivity, structure tunability and excellent stability in harsh conditions [50]. Selected publications on the development and application of carbon-based nanomaterials for advanced water treatment are listed in Table 1.

Single and multiwalled carbon nanotubes (CNTs) can be employed as catalytic supports and they are characterized by excellent properties (moderate-high specific surface area and electronic conductivity, minimum leaching of the active phase, thermal, chemical and mechanical stability in extreme environments) [49]. Moreover, CNTs are qualified as efficient nanoadsorbents for the removal of many kinds of water pollutants (heavy metals, organic pollutants, etc.) and may be functionalized with numerous ways for the improvement of their characteristics [51,52]. The catalytic activity of inert carbon nanomaterials can be enhanced by different approaches including introduction of intrinsic defects, doping with heteroatoms and the adsorption of organic molecules [52]. CNTs can be fine-tuned to enhance their abatement efficiency against a specific pollutant by using different chemical, physical, biological and/or mechanical approaches [9]. Owing to their unique characteristics, and in particular their high adsorption capacity, they are favorable nanoadsorbents for pharmaceuticals and personal care products, with π–π and n–π interactions thought to be the principal mechanisms for the abatement of organic compounds. Moreover, hydrogen bonding has a significant role in the adsorption of organic compounds on carbon based materials, and also Lewis acid-base interactions may facilitate their high adsorption [53]. As it concerns their antimicrobial properties, severe damage of cell membrane and contact of CNTs with bacterial cells has been identified as one of the main inactivation mechanisms; oxidation stress is another possible mechanism of action [54].

Graphitic carbon nitride (g-C_3_N_4_) is a well-recognized photocatalyst consisting of low-cost, earth-abundant elements. It is a “metal free”, visible light induced semiconductor with interesting characteristics, including large specific surface area, high adsorption capacity, good electronic properties, no metal dissolution and no nitrogen loss [55,56]. Due to its exceptional thermal stability and acid–alkali resistance, it is recognized as the most stable allotrope of carbon nitride under environmental conditions [57]. Different approaches have been used to enhance its photocatalytic activity (noble metal deposition, hybridization, mesoporous creation, metal modification and non-metal dropping) and different applications have been reported for the abatement of water pollutants [56]. Interestingly, pristine g-C_3_N_4_ has very good antibacterial and antiviral activities under visible-light irradiation, supporting its application in water disinfection [55]. In their review, Chauhan et al. (2018) presented the gC_3_N_4_/ZnO nanocomposite as an example of heterojunction-type photocatalytic system. The movement of the excited electrons from the higher energy potential CB of g-C_3_N_4_ to the lower energy potential CB of ZnO, results in the degradation of the pollutants by reduction. Moreover, the transfer of holes from the VB of ZnO to the VB of g-C_3_N_4_ facilitates the restrain of hole oxidation [53].

Section 2.2 has been dedicated to the presentation of an interesting group of carbon materials with exceptional characteristics that is graphene-based composites.

### 2.2. Graphene Based Composites

Graphene as well as graphene oxide (GO) have attracted significant interest worldwide, during the last decades. Graphene is a new allotrope of carbon and is characterized by a 2-dimensional honeycomb crystal structure of sp2 bonded carbon atoms. Graphene possesses unique properties such as large specific surface area, super charge carriers’ mobility, outstanding electrical conductivity, high thermal conductivity, ideal mechanical strength, high adsorption capacity and exceptional optical properties [63]. It is usually coupled to other photocatalytic nanomaterials, e.g., TiO_2_, and finds application in the degradation of environmental pollutants [64,65]. GO is a highly oxidized form of graphene with different oxygen functionalities (hydroxyl, phenol and epoxy groups mainly at the basal plane and carboxylic acid groups at the edges). It is hydrophilic due to its significant content of oxygen-groups and has unique properties [66,67]. It can be easily mixed with various materials, forming nanocomposites with enhanced properties. It can act as a good support for the synthesis of metal/metal oxide nanoparticles, and as reinforcement agent for the preparation of nano composites with different polymers, for the photocatalytic degradation of wastes from aqueous media [64,68]. Highly delocalized π-electrons, contribute to the graphene/graphene oxide potential for the degradation of pollutants. A significant rate of adsorption to the graphene sheets because of high π–π interaction between aromatic rings on adsorbed pharmaceuticals and graphene, has been reported [53]. Table 2 summarizes selected publications on graphene based composites for the treatment of emerging water micropollutants and pathogens.

Fahiminia et al. first described the phytosynthesis of copper nanoparticles (Cu NPs) reduced graphene oxide (rGO) nanocomposite (Cu/rGO), its efficiency in the degradation of 4-nitrophenol in aqueous media and its enhanced reusability characteristics [69]. In the study of Huang et al., a series of graphene supported p–n heterojunction rGO@Cu_2_O/BiVO_4_ composites were synthesized with different Cu_2_O doping showing a high photocatalytic activity for the simultaneous Cr(VI) reduction and sulfamethoxazole oxidation under LED light at neutral pH, providing a method for the treatment of coexisting heavy metals and antibiotics wastewater contaminants [70]. Graphene modified anatase/titanate nanosheets (G/A/TNS) were used by Liu et al. for the solar light-driven photocatalytic degradation of sulfamethazine, yielding 96% degradation at 4 h, good reusability and enhanced photocatalytic activity compared with the neat anatase and unmodified anatase/titanate nanosheets (A/TNS) [71]. Peng et al. evaluated the potential of a Fenton-like system Fe^3+^/H_2_O_2_ for the degradation of triclosan in the presence of GO. They reported that triclosan could be destroyed efficiently up to 90% after 30 min. Moreover, degradation byproducts of lower toxicity were produced, compared with parent compounds [67]. The effect of GO’s photoactivity on bisphenol A (BPA) was studied by Adeleye et al. who showed that the degradation of BPA was mainly due to the oxidative potential of the valence band holes produced during solar irradiation of GO [66]. Park et al. demonstrated the effective degradation of perfluorooctanoic acid by immobilized and reusable photocatalyst using graphene oxide deposited TiO_2_ nanotubes array (GO/TNA) on Ti sheet [72]. Reduced graphene oxide nanosheets decorated with Au–Pd bimetallic alloy nanoparticles were fabricated by Darabdhara et al. and were found to completely degrade phenol after 300 min and its derivatives 2-chlorophenol and 2-nitrophenol after 18 min, under natural sunlight in aqueous medium [64].

### 2.3. Metal Oxides and Composites

Metal oxide nanoparticles represent a field of materials chemistry that attracts considerable interest because of their numerous applications in many fields, including catalysis and environmental remediation [78]. TiO_2_ antimicrobial properties are due to the formation of reactive oxygen species (under UV/visible irradiation), which destroy the cellular membrane, DNA and proteins, disrupt electron transfer, affect the respiration process and release toxic ions. The formation of O_2_^−^ on its surface or the adsorption of negatively charged bacteria/spores on its positively charged surface is on the base of the disinfection properties of MgO. ZnO is characterized by a large band gap and the application of photon energy greater than the band gap results in holes (h^+^) and free electrons formation and the final production of H_2_O_2_ [54].

Akhil et al. studied ZnO NPs capped with different capping agents (e.g., gelatin, ethylene glycol (EG), polyvinylpyrrolidone (PVP) and polyvinyl alcohol (PVA)), which were prepared by a chemical coprecipitation method. The photocatalytic activity of the NPs (uncapped and capped) was assessed for the degradation of methylene blue under visible light. ZnO-PVA had the lowest photocatalytic activity followed by ZnO-G, ZnO-EG, ZnO-PVP and ZnO NPs. The studied particles were also tested for their antibacterial and antibiofilm activities against *Staphylococcus aureus* and *Pseudomonas aeruginosa* [79]. The fabrication of photocatalytic nanocomposite of maghemite nanoparticles coated with silica and carbon doped titanium dioxide, by a stepwise approach via coprecipitation and sol–gel methods, was reported by Mungondori et al. The nanocomposite could efficiently degrade organic pollutants and was magnetically recoverable [80]. Trawiński and Skibiński reported recently on a multivariate comparison of thirteen nanostructured metal oxides (Bi_2_O_3_, CeO_2_, NiO, Co_3_O_4_, Fe_2_O_3_, SrTiO_3_, Pr_6_O_11_, WO_3_, SnO_2_, TiO_2_, ZnO, ZnFe_2_O_4_ and ZrO_2_) using a model mixture of 26 psychotropic pharmaceuticals in river water, under simulated solar irradiation [81]. A hydrothermal method was used by Fakhri et al. to fabricate ZnO quantum dots decorated CuO nanosheets and TiO_2_ quantum dots decorated WO_3_ nanosheets composites, which were shown to photocatalytically degrade (up to 75%) two neurotoxin compounds under UV light and UV light coupled with microwave irradiation. Additionally, both nanosheets presented antibacterial activity against *Enterococcus faecalis* and *Micrococcus luteus,* good reusability and photocorrosion inhibition properties [82]. Islam et al. reported on the fabrication of quasi-0D/2D ZnO/MoS2 highly-efficient visible-light responsive nanocomposite photocatalysts; the authors demonstrated the excellent photocatalytic efficiencies of the nanocomposites under visible light in comparison to bare ZnO NPs, pristine MoS2 nanosheet and the commercial P25 catalysts [83]. Huaccallo et al. reported on the preparation of a magnetic magnetite/multi-walled carbon nanotube (Fe_3_O_4_/MWCNT) catalyst for the degradation of diclofenac, i.e., 95% after 3 h. Real aqueous matrices (a wastewater treatment plant effluent, surface water and hospital wastewater) were also used to evaluate the performance; 60% TOC removal occurred after 8 h of treatment, and this was accompanied by substantial ecotoxicity reduction [49]. Liu et al. reviewed magnetic nanomaterials, emphasizing the excellent magnetic and chemical stability properties of metal oxides MFe_2_O_4_ NPs (M = Fe, Co, Mn, etc.) [59]. The efficacy of Ag@TiO_2_ nanoparticles, in both free and immobilized form, for the photocatalytic disinfection of water contaminated with *E. coli* under UV light irradiation, was shown by Sreeja et al. Complete disinfection of 40 × 10^8^ CFU/mL was achieved in 60 min under UV irradiation with 0.4 g/L catalyst loading, proving the superior photocatalytic disinfection characteristics compared to TiO_2_ nanoparticles. Moreover Ag@TiO_2_ was shown to be effective (59.3%) for the degradation of bacterial endotoxin by UV photocatalysis [84]. Santiago et al. used three magnetic materials (magnetite (Fe_3_O_4_), as well as the respective core–shell magnetic silica (SiO_2_@Fe_3_O_4_) and rGO-Fe_3_O_4_ (rGO-MN)) as supports to prepare multi-component catalysts using a specific TiO_2_ material (EST-1023t). The photocatalytic activity was assessed for the degradation and mineralization of the fungicide imazalil in both deionized water and a synthetic wastewater. Compared to bare TiO_2_, the magnetic catalyst showed a higher photocatalytic activity [85].

The photocatalytic treatment of kraft pulp mill effluent and mining effluent with nano-TiO_2_ or nano-Fe_2_O_3_ was reported by Nogueira et al. Experiments were performed under UV light with or without hydrogen peroxide. Both catalysts were capable of significantly reducing color, COD, aromatic compounds and the toxicity of the mill effluent. Regarding the mining effluent, significant reductions of toxicity and of three out of seven metals present in the wastewater, were achieved [86]. In another study, Nogueira et al. assessed the effect of coupling photocatalysis (nano-TiO_2_/UV, nano-Fe_2_O_3_/UV, nano-TiO_2_/H_2_O_2_/UV and nano-Fe_2_O_3_/H_2_O_2_/UV) with biological treatment with fungi for the reduction of the toxicity and the improvement of the characteristics of olive oil mill wastewater. This approach was generally effective in removing COD, total phenolic content (TPC) and ecotoxicity, while the most promising system comprised pretreatment with nano-Fe_2_O_3_/UV followed by biological treatment with *P. chrysosporium* [87]. The capacity of iron oxalate-based magnetic catalysts for the photodegradation of bisphenol A was demonstrated by Neamtu et al.; a PEGylated magnetite modified with iron (II) oxalate was the most active material, leading to complete degradation in ca 15 min of reaction [88]. 

### 2.4. Metal Organic Nanomaterials

Metal organic frameworks (MOF) based composites (e.g., with metal oxide/sulfides, noble metal NPs, GO, etc.), are a new class of catalysts, which may be applied for the degradation of organic pollutants and environmental remediation [89]. The composite materials have superior properties, compared with those of their individual components. They are porous solids of highly designable, ordered and crystalline nature, composed of coordination bonds between metal ions and organic ligands, characterized by large internal surface areas, thus with a significant potential to incorporate a large number of accessible catalytic sites [90,91,92,93,94]. The pores of these materials can be fine-tuned in order to obtain the desired properties due to the elevated tunability of their synthesis. MOFs have the potential to combine the advantages of homogeneous catalysis (e.g., well-defined structure and/or ligand environment) with those of heterogeneous catalysis (e.g., site isolation and/or recyclability). It has to be noted that more than 20,000 MOFs have been reported until today. Some of them are characterized by exceptional catalytic performances, which are generally implemented by metal node, functional organic linker or both [90]. Sharma et al. reviewed the environmental applications of MOFs with emphasis on the performance enhancement of advanced oxidation processes. The application of MOFs for the abatement of water pollutants involves adsorption and advanced oxidation processes. Adsorption mechanisms are mainly based on hydrophobic, electrostatic, acid–base π–π interactions and hydrogen bonding. As it concerns the MOF-catalyzed advanced oxidation processes, the principal photocatalytic mechanisms are the ligand-to-metal charge transfer and the excitation of metal-oxo clusters in MOFs by adsorbing incident photons (under UV and/or visible light irradiation) [95]. Recently, Rasheed et al. (2020) summarized the advancements of MOF-based engineered materials (i.e., pristine MOFs, MOF derivatives or MOF composites) for photocatalysis, electrocatalysis and biocatalysis [96], while Rojas and Horcajada (2020) focused on the progress in the applications of MOFs in the elimination (adsorption and/or degradation) of emerging organic pollutants from water [97].

The main concern about MOFs and MOF-related materials is their use in real world applications. Usually, MOF-based catalysts are employed as ideal models for structure–property relationship laboratory studies but large-scale synthesis of low-cost MOFs are needed for practical applications. The coordination interaction between the metal and the ligand in MOFs is not as stable as those in other materials (active carbons or zeolites), which would lead to the collapse of the framework during reactions. More stable MOFs have been fabricated until today, with optimized resistance to water, acid, base and other harsh conditions, but still their stability cannot be compared to that of other inorganic or organic porous materials such as those mentioned before (zeolites, mesoporous silica and porous carbons) [94,98]. Due to some drawbacks related to insolubility, brittleness, molding difficulty and restricted compatibility with other materials, new fabrication methods of MOFs should be developed to support their application in specific devices [99]. Due to their significant tunability, MOFs and MOF-based materials are expected to have a bright future of evolution and applications [98].

Table 3 summarizes selected publications on MOFs-based nanomaterials for the treatment of emerging water micropollutants and pathogens.

### 2.5. Commercially Available Nanomaterials

Various nanomaterial-based commercially available products for water purification are already available in the market (Karofi, Lifestraw, Tupperware). Nanomaterials-based membranes, 2D graphene nanomaterials, different nanocomposites and titanium-based, iron, zinc oxide and silver nanoparticles, have all found applications in water purification. Nanomaterials may be used in different approaches during water purification such as nanofilters, nanofiber-based filters and nanoadsorbent [110].

Karim et al. investigated the degradation of the steroidal estrogen 17a-ethinylestradiol by commercially sourced nano zero-valent iron (nZVI) at pH 3, 5 and 7 under different oxygen conditions. Following the use of radical scavengers under nitrogen purging, direct reduction of the estrogen occurred at all pH. The radicals transforming the estrogen in the absence of purging and upon air purging were similar for a given pH, but the dominant radical varied with pH [111]. The degradation of chloridazon on the surface of UV-irradiated Zr-loaded TiO_2_ was investigated. Mesoporous TiO_2_/ZrO_2_ nanopowders prepared by the evaporation-induced self-assembly route were found to be more active than P25 TiO_2_ [112].

Perazzoli et al. investigated the effect of commercial magnetite nanoparticles (Fe_3_O_4_-NPs) on ammonium-oxidizing bacteria activity during wastewater treatment. Iron oxide NPs are known to possess a significant commercial potential because of high catalytic activity, antimicrobial activity and magnetic properties. The authors reported that the Fe_3_O_4_-NPs concentration, which reduced the NO_2_^−^-N production rate by 50%, was 0.483 g/L. Moreover, NPs were located in the sludge indicating the possibility of environmental dissemination through biomass disposal [113]. The simultaneous mineralization of five emerging organic contaminants, which were dissolved in deionized and tap water was performed by UV-photocatalytic degradation on commercial TiO_2_ micropearls and this was compared against TiO_2_ nanolayer on quartz wool. The extent of mineralization exceeded 90% in deionized water and was approximately 70% in tap water after 4 h of treatment, while the catalyst was reused for seven cycles without significant efficiency loss [114]. Xu et al. reported that TiO_2_ nanowires on flexible PET fabrics were found to possess higher photocatalytic activity towards degradation of rhodamine B in water under UV light than either TiO_2_ nanotube array or commercial P25 TiO_2_ nanoparticulate films on metallic Ti substrates. Interestingly, a significant disinfection potential against *E. coli* and *S. epidermidis* was also recorded under visible light [115]. Triantis et al. studied the degradation of a cyanotoxin (microcystin-LR) using a nitrogen doped TiO_2_ (N-TiO_2_) photocatalyst, under UVA, solar and visible light, in comparison to commercial P25 TiO_2_, Kronos and reference TiO_2_ nanopowders. Under UVA irradiation, P25 TiO_2_ and N-TiO_2_ exhibited comparable activity yielding almost complete cyanotoxin degradation after 20 min, while under solar light, N-TiO_2_ had comparable photocatalytic activity to P25 and Kronos TiO_2_. Finally, under visible light, the N-TiO_2_ photocatalyst had significant photocatalytic efficiency in contrary to P25, which did not show any response [116]. The removal of the s-triazine herbicide terbuthylazine from aqueous solution by various treatment methods has been reported by Álvarez et al. The methods included adsorption onto activated carbon and multiwalled carbon nanotubes (MWCNT), UVC/H_2_O_2_, UVC photolysis, single ozonation, O_3_/H_2_O_2_, catalytic ozonation (activated carbon, MWCNT and TiO_2_ as catalysts) and solar driven processes such as TiO_2_ photocatalytic oxidation and photo-ozonation. The herbicide could be removed effectively by activated carbon adsorption, as well as solar photocatalysis with P25 TiO_2_ and laboratory prepared anatase TiO_2_ nanoparticles [117]. The catalytic activity of nanostructured TiO_2_ crystals synthesized by gel to crystalline conversion was found to be 2.6 times higher than that of commercial P25, with complete photoinactivation of *E. coli* being achieved within 60 min under irradiation at 400 nm. Moreover, the photoactivity of nanotitania was enhanced by 1.6 times when the source of irradiation shifted from 360 to 400 nm in contrary to P25, which showed no change [118]. Su et al. reported that the amorphous triple-shelled Ag@Fe_3_O_4_@SiO_2_@TiO_2_ hierarchical nanospheres they synthesized were more active than commercial P25 TiO_2_, pure TiO_2_ microspheres, Fe_3_O_4_@SiO_2_@TiO_2_ and annealed Ag@Fe_3_O_4_@SiO_2_@TiO_2_ nanocomposites for the photodegradation of organic contaminants and photoreduction of toxic heavy metals in wastewater, under visible light, Xe lamp and sunlight. Additionally, the authors underlined that the excellent chemical stability, magnetic recyclability and accessible synthetic route could make these multifunctional photocatalysts promising candidates for water treatment applications [119]. The photocatalytic properties of Ag_3_PO_4_ composites with combustion synthesized TiO_2_ and P25 TiO_2_ under sunlight were compared against each other in the study of Eswar et al. The enhanced photodegradation of bacteria in comparison to the commercial TiO_2_ nanocomposite was attributed to extended light absorption, better charge transfer mechanism and higher generation of hydroxyl radicals and valence band hole [120].

Numerous nanomaterial-based commercially available products for water purification are available currently. Interestingly, approximately 53% of the silver containing products registered with USEPA contained silver in the nanoform. Companies such as Seldon Laboratories, Inc., Panasonic, Karofi, TATA, Sciessent LLC, Katadyn Asia Inc. have commercialized products based on nanosilver. Other water purification commercial products of Panasonic and Nano Sun have developed products based on nano TiO_2_, while products of NanoH_2_O, Agronide corporation, SolmeteX Inc. and NASA were based on super hydrophilic nanoparticles, nano alumina fibers, iron oxide NPs and carbon nanotube, respectively [110].

## 3. Inactivation of Microorganisms

Waterborne diseases are those transmitted through the ingestion of contaminated water, when water acts as a passive carrier of the infection agent. The most common cause is fecal pollution of human and/or animal origin. Bacteria, viruses and protozoa are the cause of many emerging and new waterborne infectious diseases. It is well known currently that classic bacterial indicators of fecal contamination (*Escherichia coli* and enterococci) are not adequate to indicate the presence and concentration of viruses and protozoa [121].

### 3.1. Bacterial Indicators and Pathogens

Bacterial agents including *E. coli, Pseudomonas, Shigella, Salmonella, Mycobacterium avium, Vibrio cholerae*, *Campylobacter, Helicobacter*, *Legionella*, etc., have been linked to waterborne diseases, and are considered emerging and/or re-emerging. 

The antibacterial activity of zinc oxide nanoparticles has been well-documented. Akhil et al. showed almost 100% inhibition of *S. aureus* and *P. aeruginosa* at 100 mg/L of ZnO NPs [79], while Raghupathi et al. documented a similar finding for the inhibition of *S. aureus* at 5 mM of ZnO NPs [122]. Higher inactivation activity for *E. coli* and *S. aureus* induced by DOX-ZnO/PEG nanocomposites has been reported under visible light irradiation [123]. ZnO NPs have been also shown to reduce *S. aureus* [79,124], *P. aeruginosa* [125,126] and *E. coli* [124] biofilms, an effect, which could be attributed to the generation of reactive oxygen species. Rich bibliographic data also exist on the antimicrobial activity of silver nanoparticles [127,128,129]. Biofilm formation of methicillin resistant *S. aureus* was found to be inhibited by silver nanoparticles (AgNPs) [128]. AgNPs of 10 nm were found to have increased antimicrobial activity against *Methylobacterium* spp. probably due to the release of silver ions. Interestingly, the antimicrobial activity of 10 nm particles was higher than that of the 100 nm ones, with the same concentration and a similar surface chemical composition [129]. AgNPs and slowly released silver ions have been also shown to inactivate *Gordonia* sp., a bacterial group with beneficial effects in natural water bodies and wastewater treatment. The highest dosage of AgNPs resulted in the greatest average log inactivation for *Gordonia* sp. [127], similarly to what was recorded with increased dosage of AgNPs (from 1 to 8 mg/L) against *E. coli* [130]. A 99.9% *E. coli* inactivation within 30 min under solar light was achieved by an Ag/TiO_2_ nanofiber membrane [131]. 

The degradation of organic compounds and the bacterial disinfection (*E. coli*, *S. typhimurium* and *S. sonnei*) in water by heterogeneous photocatalysis with TiO_2_ and near-neutral photo-Fenton were less efficient when the two agents were simultaneously present [41,132]. The disinfection potential of TiO_2_, Pt-TiO_2_ and Ag-TiO_2_ photocatalysts against *E. coli* was studied by Suri et al. under artificial light and sunlight and the Ag-TiO_2_ photocatalyst was found to be more effective in both water and wastewater matrices [133]. The Ag–TiO_2_ nanocomposite was shown to possess an enhanced antibacterial activity against *E. coli*; 1g/L of the nanocomposite resulted in 99.99% inhibition of the bacterial growth [134]. Ti/TiO_2_–Ag coupled into a photoelectrocatalytic process was found to be a promising antimicrobial tool, achieving 100% inactivation of *Mycobacterium smegmatis*, under UV irradiation for 3 min [135]. Photocatalytic disinfection of water by Ag core and TiO_2_ shell (Ag@TiO_2_) nanoparticles under UV irradiation was assessed by Sreeja and Vidya Shetty, with nanoparticles being both in their free and immobilized form in cellulose acetate film. During the photocatalysis treatment, both *E. coli* disinfection and degradation of endotoxins occurred. The authors underlined the potential for large-scale application in drinking water treatment plants, as well as in household purification units [84].

A significant antibacterial activity against *K. pneumonia* and *E. coli* was recorded by Venieri et al., who used Co- Mn- and binary Mn/Co doped TiO_2_ catalysts under solar light [136]. Fakhri et al. fabricated ZnO quantum dots decorated CuO nanosheets and TiO_2_ quantum dots decorated WO_3_ nanosheets composites; they showed that enhanced photocatalytic degradation of neurotoxins, as well as inactivation of gram positive and gram negative bacterial strains, *Enterococcus faecalis* and *Micrococcus luteus* could be achieved [82]. The inhibition of bacterial growth through the electrostatic interaction of negative bacterial charges and the positive metal oxide nanostructures was suggested to be the main reason of the antimicrobial activity. Significant antibacterial activity (reaching 100% within 15 min of visible light irradiation) against *E. coli* and *S. epidermidis* was recorded using TiO_2_ nanowires on flexible PET fabrics. Characteristics such as phase junctions, abundant surface hydroxyl groups, the narrowed band gap of 2.5 eV due to the nitrogen doping and the unique 1D nanostructures were thought to be responsible for the enhanced antibacterial and photocatalytic activity of the material [115]. A novel nanoporous copper aluminosilicate (CAS) material was prepared by Hemdan et al. The antimicrobial action and minimum inhibitory concentration of this promising material were determined against different target microorganisms (*Escherichia coli, Salmonella enterica, Pseudomonas aeruginosa, Listeria monocytogenes, Staphylococcus aureus, Enterococcus faecalis, Candida albicans* and *Aspergillus niger*). The authors found that complete inactivation was achieved within 20–40 min against all tested microorganisms and underlined the potential use of nanoporous CAS for water disinfection [137]. Domínguez-Espíndola et al. showed that complete fecal coliform bacteria inactivation could be achieved in real urban wastewater within 6 min of UV irradiation using nanoparticulated films of TiO_2_ and TiO_2_/Ag (4% w/w). Moreover, the authors proved that the principal reason of bacterial inactivation was the cell wall rapture [138]. A new method for the synthesis of nano-photocatalysts based on colloidal nanocarbon–metal composition (NCMC) with titanium as a metal has been described by Khaydarov et al. These nano-photocatalysts were successfully used to destroy *E. coli* bacteria in 10–30 min in water [139]. In the study of Pandiyan et al. SnO_2_-doped nanocomposites (with SnO_2_ being the dopant in sulphonated GO and CNT), showed significant dose-dependent bactericidal activity under visible light, against *E. coli* and *P. graminis* isolated from a domestic wastewater treatment plant (Pandiyan et al. 2019). The significant antibacterial activity of SnO_2_-doped nanomaterials against a wide variety of bacterial species (*E. coli*, *P. aeruginosa*, *S. aureus*, *L. monocytogenes*, *B. subtilis*, *S. typhi* and *T. viride*) was previously reported [140].

Zhan et al. developed a simple method for the rapid removal of pathogenic microorganisms (both bacteria and virus) from water, using modified core–shell Fe_3_O_4_–SiO_2_–NH_2_ nanoparticles. The removal efficiencies for Gram positive (*S. aureus* and *B. subtilis*) and Gram negative bacteria (*E. coli*, *P. aeruginosa* and *Salmonella*) were 93.4%, 97.4%, 95.1%, 90.1% and 90.1%, respectively. Interestingly, the nanoparticles were shown to effectively isolate pathogens from real river samples with efficiencies ranging between 92% and 96.3% [141].

Jin et al. used the antibacterial agent, cetyltrimethylammonium bromide (CTAB), in order to modify Fe_3_O_4_ nanoparticles and to produce bactericidal paramagnetic nanoparticles (Fe3O4@CTAB). The authors claimed that these nanoparticles have a great potential in water disinfection and showed that more than 99% of *E. coli* and *B. subtilis* bacteria could be inactivated within 60 min [142]. SnO_2_/PSi/NH_2_ nanocomposite has been shown to possess a significant antibacterial potential toward *E. coli* and *S. aureus*. The nanoadsorbent was produced using SnO_2_ nanoparticles as the core, coated with mesoporous silica and modified with 3-aminopropyl triethoxysilane [143].

Hassouna et al. focused on the antibacterial effects of kaolin clay and its loaded forms with carbon nanotubes and silver nanoparticles. They studied bacterial strains derived from different water matrices (surface, underground, tap water and wastewater). They found that AgNPs-loaded clay (at 0.1 mg/L) showed the most pronounced antibacterial efficacy against *Salmonella* spp (90%), *E. coli*, *Klebsiella pneumonia* and *Shigella flexneri* (80%) and *Klebsiella aerogenes* (70%) after 2 h of exposure time [144]. The antibacterial activity of CNTs-loaded clay at the same concentration was 70% for each of *Salmonella* spp. and *Klebsiella pneumonia*, while it was 60% for *E. coli* strains [144,145]. In another study, hybrid polyaniline/graphene/carbon nanotube materials were fabricated and achieved 99.5% and 99.2% removal for *S. aureus* and *E. coli*, respectively [146].

Extensive research has focused on the antimicrobial effects of g-C_3_N_4_-based photocatalysts for water disinfection. The antibacterial activities of BiVO_4_ QDs/g-C_3_N_4_ and AgVO_3_ QDs/g-C_3_N_4_ have been shown to reach 87.5% and 96.4% against *Salmonella*, respectively. The antimicrobial effect was achieved within only 10 min under visible light irradiation [147]. In another study, the antibacterial effect of GO/g-C_3_N_4_ was reported [148]. It was found that significant *E. coli* inactivation (97.9% of 10^7^ CFU/mL) could be achieved under visible light within 2 h. Metal-free antimicrobials have been proposed as ideal candidates for water disinfection and microbial control [52].

### 3.2. Viruses

The enteric viruses of human stool and urine belong to more than 140 types [149]. Most of the viruses transmitted via the fecal–oral route are non-enveloped and are characterized by high environmental stability, including significant emerging and re-emerging etiological agents. Interestingly, current methods of wastewater treatment are not effective for the abatement of these viruses, which are released from treated and untreated wastewater. The most relevant human pathogenic viruses belong to the families of *Caliciviridae, Adenoviridae, Hepeviridae, Picornaviridae* and *Reoviridae* [121]. Pathogenic enteric viruses are characterized by extremely small size (e.g., 23–25 nm for MS2, 28–30 nm for hepatitis A virus, 27–30 nm for poliovirus, 65–85 nm for adenovirus and 35–39 nm for norovirus) and their presence in environmental waters pose a significant risk to human health. Coliphages MS2 and ΦX174 have been studied as indicators of viral pollution in fecally contaminated water along with adenoviruses. However, experimental data based on the use of coliphages should be interpreted with prudence, since they cannot predict the behavior of numerous different types of viruses. In our review, the vast majority of the studies used MS2 phage as viral indicator, while limited studies used Poliovirus-1.

In the study of Cheng et al., commercial nano P25 TiO_2_ was found to be more effective for MS2 phage removal (which was used as model virus) than numerous other nanomaterials (carbon nanotube, graphene, nano ZnO, nano Ni, nano Fe_3_O_4_ and nano TiO_2_–anatase) [150]. Removal efficiency was found to increase with increasing P25 concentration in the range of 0–1000 mg/L, while higher concentrations had no effect. Under certain conditions (P25 concentration, irradiation dose and transmembrane pressure), a removal efficiency of up to 100% could be achieved for MS2. These data support the findings that P25 has advanced adsorption and photocatalysis performance, compared to other nanomaterials, and that the nanomaterial–membrane coupling system is ideal for virus removal. The enhanced disinfection potential of Cu–TiO_2_ nanofibers for bacteriophage f2 and its host *E. coli* 285 with visible-light photocatalysis was proved by Zheng et al. As expected, the virus was characterized by higher resistance to photocatalytic oxidation than bacteria, viral disinfection was affected in virus/host bacteria mixed system and reactive oxygen species were crucial for virus inactivation [151]. Zhan et al. achieved capture efficiencies over bacteriophage f2 and Poliovirus-1 of 76.7% and 81.5%, respectively, using amine-functionalized magnetic Fe_3_O_4_–SiO_2_–NH_2_ nanoparticles [141]. Liga et al. showed that silver doping of TiO_2_ nanoparticles was an effective way to increase TiO_2_ photocatalytic activity for virus inactivation; this was assessed for the inactivation of bacteriophage MS2 in aqueous media [152]. The enhanced virucidal performance of g-C_3_N_4_ against MS2, compared to other visible light active photocatalysts such as Ag@AgCl, Bi_2_WO_6_ and NeTiO_2_ was reported elsewhere [153]. It was found that 8-log of MS2 were inactivated without regrowth after 6 h under visible light; following treatment optimization, this time could be reduced to 4 h, supporting the potential application of g–C_3_N_4_ for water viral disinfection. The g–C_3_N_4_/EP composite (carrier of expanded perlite-EP) resulted in complete inactivation of 8-log *E. coli* and MS2 within 3 and 4 h of visible light irradiation, respectively. Interestingly, viruses of real source water samples could be inactivated without regrowth after 7 h. All these findings underlined the antimicrobial potential of water-surface floating photocatalytic composites for source water disinfection [55].

### 3.3. Protozoa

Protozoan parasites are among the most important waterborne pathogens, with many emerging and re-emerging members including *Cryptosporidium, Giardia, Cyclospora, Acanthamoeba*, *Isospora*, etc. Most of the reviewed studies were focused on *Cryptosporidium parvum*.

Abebe et al. studied the independent effects of silver salt and nanoparticles on *Cryptosporidium parvum* and the removal of this protozoan pathogen of global significance to human health by physical filtration in porous ceramic filter media. They found that the removal efficiencies ranged from 96.4% to 99.2%. They showed that physical filtration and silver nanoparticle disinfection contributed to treatment of *C. parvum* using silver impregnated ceramic water filters, although the contribution of physical filtration was likely greater than silver disinfection [154]. In another study, Darwish et al. fabricated a composite by embedding silver nanospheres onto aragonitic cuttlefish bone (CB)-stabilized samarium doped zinc oxide (Sm-doped ZnO) nanorods. They showed that Ag@Sm-doped ZnO/CB presented significant biocidal efficiency against pathogenic bacteria and parasites in dark- and photo-conditions. In particular, the nanocomposite exhibited enhanced disinfection efficiencies for *Staphylococcus aureus* (80%), *Pseudomonas aeruginosa* (60%) and *Schistosoma mansoni* cercariae (100%) linked with progressive demolition in cercarial body. Moreover, the synthesized nanocomposite also possessed exterminating action against *Schistosoma mansoni* adult worms serving near 100% worm-mortality accompanied by significant disintegration of worm body [155]. Recently, Hussein et al. demonstrated that MgO NPs had a significant effect against *Cyclospora* oocysts. Different doses of MgO NPs (1.25–25 mg/mL) were used and the anti-*Cyclospora cayetanensis* effect on both unsporulated and sporulated oocysts was shown [146]. Sunnotel et al. focused on the disinfection of surface water, which was contaminated with *Cryptosporidium* oocysts, by TiO_2_ photocatalysis. They found that photocatalytic inactivation of *C. parvum* oocysts occurred in both buffer solution (78.4% after 180 min) and surface water (73.7% after 180 min). No significant disinfection occurred in the absence of TiO_2_ in the dark or under UVA irradiation [156].

## 4. Performance Limitations

### 4.1. Toxicity

Although nanomaterials are valuable tools for water treatment technologies, their synthesis routes usually involve the use of hazardous and volatile chemicals, thus creating a significant secondary pollution [1]. One of the main limitations when using metal and metal oxide NPs is the possible toxicity of these materials, along with their byproducts and the costs linked to their recovery [2]. What should be underlined is that biological systems have not been evolved with the engineered nanomaterials, which are currently produced and continuously released in the environment. Thus, various engineered nanomaterials have been shown to exert a wide range of ecotoxicological effects on different organisms (bacteria, plants, invertebrates, fish, etc.) [7]. Aravantinou et al. studied the effect of zinc oxide nanoparticles on freshwater and marine microalgae cultivated in different media. Significant differences were recorded on microalgae growth rates, with the marine being more sensitive than the freshwater species. ZnO NPs were shown to have toxic effects in all species tested, depending on the time of exposure, species type, NPs concentration and mainly the used culture medium [157,158]. Exposure to NPs has also been linked to mucosal and peribronchial inflammation and irritation of the skin in humans [159]. 

Ideally, toxicity studies should focus on different organisms of the food chains/pyramids. However, most of the studies are limited to selected representatives. Moreover, they use unrealistic concentrations of pristine NPs and not functionalized ones. The study of NPs in the form in which they are really released in the environment, e.g., embedded in a matrix and/or are finally being exposed to living organisms (e.g., after chemical and/or biological modifications due to numerous environmental factors) is important for a realistic assessment of their effects [7,160]. Ironically, the same properties of nanomaterials that make them useful, that is molecular structure of nanomaterial constituents and size, are also linked to their potential toxicity. Toxicity risks may be reduced by immobilization of the nanoparticles on support media or reactor surfaces, an approach that could also have positive effects, namely improved activity and reduced aggregation [161]. The control of the dissolution by stabilizing coatings is an effective approach for nanoparticles such as nano-Ag, which release toxic metals. The size and shape of NPs can be also optimized. To be on the safe side, it is always preferable to use NPs with non-hazardous constituents. Alternatively, barrier strategies should be applied, such as membranes and magnetic separation in order to avoid their release and effectively recover them [162].

CNTs have been linked to the damage of different organs, as well as DNA impairment in the human body. The exposure to MWCNTs has not been linked to cancer, however significant cytotoxic, genotoxic and apoptotic effects have been recorded. Moreover, the negative effects of CNTs on microbial communities, aquatic organisms and mammalian systems have also been demonstrated, thus underlying the need of extensive investigations before their use as adsorbents in wastewater treatment [9]. Fullerenes have also been shown to exert ecotoxic effects to bacteria, daphnia, earthworms, fish and human cell lines, while human cytotoxic and hemolytic effects have been proved for other complex nanocompounds [163]. Short and long terms assessment of the in-vitro and in-vivo toxicity of nanomaterials has to be performed [54].

One of the conclusion remarks of the review of the NEREUS COST Action ES1403, was that since the application of ozonation or advanced oxidation processes for the abatement of emerging pollutants in wastewater, results in the production of oxidation transformation products with potential biological effects, ecotoxicological studies have to be performed. Although post-treatment steps (e.g., with sand filters) are effective to face this problem after ozonation, the treatment costs are increasing [14]. In their review, Gavrilescu et al. (2015) commented on the application of modern technologies such as DNA microarrays in an emerging field of ecotoxicology, the ecotoxicogenomics. They also stressed out the development of molecular biomarkers as detection tools for the investigation of the bioavailability of nanoparticles in the environment [19]. Nanotoxicology data showed no nanoparticles to be considered as safe or non-toxic. Importantly, the study of byproducts generated through photocatalysis or ozonation processes should be performed with emphasis on their stability, concentration and physicochemical properties [53].

### 4.2. Operating Conditions

In a recent review, Guerra et al. (2018) stressed out the inherent instability of some nanomaterials under normal conditions, and thus the need to enhance their stability, prevent agglomeration, increase their monodispersity and also consider the recovery costs [2]. In another comprehensive review on the removal of pharmaceutical contaminants in wastewater using nanomaterials [53], authors recorded the operating limitations of different materials. Activated carbon was not efficient due to the presence of numerous organic compounds of natural water, which compete for the adsorption sites with pharmaceuticals, while ozonation failed due to the formation of toxic transformation products. Although advanced processes, such as catalytic ozonation, were developed to overcome the aforementioned drawbacks, the adsorption and diffusion of pharmaceutically active compounds on catalysts has become the limiting step. Despite of the progress achieved until today, a new generation of more efficient photocatalysts is still needed for better performances. As reviewed by Rizzo et al. (2019) numerous limiting factors inhibit the full-scale application and function of advanced oxidation processes for the degradation of emerging pollutants (e.g., absence of regulations for their elimination from wastewater, organic and inorganic scavengers’ presence in wastewater, variability of the effluents, etc.). Authors present among others, the advantages and drawbacks for each advanced treatment, suggesting that the decision on best technology should be made for each location taking in consideration the local conditions, the water quality from the biological treatment and the required effluent quality [14].

In natural water matrices, NPs interact physically and chemically in a complicated way, resulting in NP oxidation vs. reduction, dissolution vs. precipitation, dispersion vs. aggregation and also complexation with background chemicals [127]. Easy aggregation in, and difficult separation and recycling from water, have been identified as the main limitations of the implementation of g-C_3_N_4_ powders in water disinfection. To face the above mentioned problems, researchers loaded g-C_3_N_4_ powders on a carrier of expanded perlite [164]. The presence of a disinfection residue has a pivotal role for the control of the microbial growth during water storage and distribution. The main disadvantage of most nanomaterial-based disinfection processes is the lack of such residue [145,162]. Under normal conditions, some nanomaterials are inherently unstable and tend to agglomerate. Agglomeration is the main constrain since it can significantly affect the reactivity of the material. The prevention of the agglomeration and the enhancement of monodispersity and stability are crucial issues for an effective nanomaterial [2]. Limitations to mass transfer and elevated pressure drop have been also identified as the main drawbacks of the practical applications of powder catalysts. An approach that has been already demonstrated to face these issues is the use of structured catalysts containing carbon nanomaterials as the active phase to the process of catalytic ozonation [43]. Although the potential of CNTs as effective adsorbents for the removal of organic micropollutants from wastewater has been extensively tested, significant limitations still exist for their large scale applicability. These drawbacks could be summarized to the hydrophobicity, the elevated production cost, the purity of the produced CNTs, the problematic centrifugation separation, as well as to their release into the environment and their detrimental effects to living organisms [9]. More research efforts are also required for the comprehension and improvement of the absorption mechanisms of these materials. The significant energy and resources requirements for the production and regeneration of activated carbon are drawbacks of their application for wastewater treatment. Similarly, clogging problems consist important limiting factors of granulated activated carbon filter systems [165].

Kumar and Chowdhury (2020) have recently published a book chapter on the use of novel nanostructured photocatalysts for the environmental sustainability of wastewater treatments. They underlined the modern need of research, which has to focus on the exploitation of more stable, cost effective, efficient and industrially viable photocatalysts activated by natural light [166]. Since the work carried out so far on the development of visible light mediated photocatalytic processes for wastewater treatment is not economically feasible and environmentally friendly, the real world applications of photocatalytic reactors are very limited.

Slow reaction kinetics consist the main limiting factor for the application of TiO_2_ photocatalysis systems, although commercial treatment systems do exist [152]. Despite the numerous positive characteristics of TiO_2_ photocatalyst, some inherent disadvantages may be listed such as agglomeration, difficult recovery from treated liquid, difficulty to support powdered TiO_2_ on some materials, high recombination rate between photogenerated electrons and holes, efficient activation only by UV light due to the band gap energy of 3–3.2 eV, difficulty of significant performance improvement by loading or doping with foreign species [167,168]. To overcome the aforementioned limitations, different approaches have been tested such as addition of metals and non-metals, such as multiwall carbon nanotubes [60]. Doping, loading and sensitization of TiO_2_ have been applied to shift the light absorption towards visible light, as well as to increase the photoproduced electron-hole pairs lifetime [168]. Triantis et al. reported that their tested N–TiO_2_ nanocatalyst exhibited a remarkable efficiency for cyanotoxin destruction in the visible light, in contrary to P25 TiO_2_, which was totally inactive, thus overcoming limitations concerning UV light utilization [116]. The instability of SnO_2_ nanoparticles, which is recorded under acidic conditions, as well as the high degree of aggregation, are significant limiting factors of their adsorption potential, and thus different approaches should be used to overcome these drawbacks (combination with metal oxides, porous adsorbents, surface modification with coating materials, etc.) [143]. Although plasmonic photocatalysts such as nanostructured Au, Ag or Cu supported on metal oxides have been developed, they are still expensive approaches for the treatment of pollutants and moreover mechanistic insights are missing [166].

Pure CdS has a weak stability due to its self-photocorrosion mediated by the photogenerated holes besides the short lifetime of its charge carriers and, thus, modifications should be implemented (e.g., composting with other semiconductors or polymers, doping with metal or non-metal ions, etc.) for the improvement of its properties [77]. To overcome drawbacks of graphitic carbon nitride (g–C_3_N_4_), such as low conductivity, fast recombination and limited surface area, magnetic ferrite NPs have been combined with mesoporous g-C_3_N_4_ in order to avoid the agglomeration and deactivation of the catalyst [169]. To face the drawbacks of CNTs, i.e., (i) non-functionalized CNTs are very hydrophobic and difficult to disperse in aqueous medium, (ii) oxidized CNTs are more hydrophilic and they can be dispersed but the interaction with more hydrophobic molecules is problematic, (iii) the removal of CNTs from the medium after adsorption is difficult, (iv) loose nanoparticulated carbon is potentially toxic, Purceno et al. produced an amphiphilic composite combining carbon nanostructures, CNT and nanofibers (CNF) to be used for the adsorption of organic compounds in water [170].

Although many nanomaterials have been extensively modified and they are characterized by significant disinfection activities, they have not found real world applications; this is mainly due to the complicated methods of preparation [145,171]. To deal with the drawbacks of goethite (α–FeOOH; aggregation, difficulty in recycling, small size, fast recombination of electron-hole pairs), which limit its practical application, Su et al. developed a novel goethite anchored by graphene oxide (GO)-carbon nanotubes aerogel (α–FeOOH@GCA) nanocomposite capable of activating persulfate; the system was shown to be highly efficient for environmental applications [172]. Intrinsic weaknesses of MOFs, such as a lack of catalytically active sites and limited thermal and chemical stability, are limiting factors for their full implementation. Currently, the synthesis routes of MOFs are usually complex, time-consuming, expensive, while the scaling-up is considered a difficult process. Thus, MOF-based metal and metal oxide NP composites have been produced with enhanced characteristics. MOF composites are still under an early developing phase as heterogeneous catalysts. The innovation in the synthesis of MOFs is considered one of the future developments [89]. Novel nanotechnologies for the treatment of water and wastewater have to be socially accepted, cost effective and present no toxicity [37,162].

Immobilization of nanomaterials onto a support matrix has been applied to face the limitations of poor separation after use, blockage of sorption sites and leaching of colloidal nanomaterials [53]. In an interesting review on emerging environmental pollutants [19], authors commented on the crucial need to support research and innovation for the fabrication of innovative and economically feasible treatment technologies, in line with the uptake, mode of action and consequences of each emerging pollutant. In another review on nanotechnology for environmental applications, Guerra et al. (2018) stressed out the need to study real case scenarios and not limit the research attention at the laboratory scale, and also focus on the study of the contaminant capture and/or degradation by the applied nanomaterials; a field that is underexplored [2].

### 4.3. Reuse

Two of the most important issues linked to the cost-effectiveness of nanomaterials are recyclability and regeneration. Most of the studies consider the regeneration high if nanomaterials maintain almost 100% of their initial properties after 3–10 cycles; it should be noted that this refers to laboratory scale studies, with no economic analysis performed [163]. Although recyclability of some nanomaterials has been described, their efficacy is reduced with time, and they are finally becoming not useful [2]. Long term reusability of nanomaterials improves their cost effectiveness. Numerous studies have reported on regenerated nano-adsorbents, magnetically separable multifunctional nanomaterials and catalysts that are active through multiple reuse cycles [37,162]. The application of amino fullerene photocatalysts made with fullerene soot rather than ultrapure C60 resulted in cost savings of approximately 90% and, in parallel, showed a limited loss of effectiveness (< 10%), supporting the potential use of nanomaterials of lower purity for water treatment [161]. To improve their regeneration characteristics, several parameters should be optimized for carbon and zeolite nanomaterials, and similarly, immobilization of photocatalysts on support matrices or as in nanocomposites will favor their recovery, reusability and economic feasibility [53].

## 5. Future Trends

Emerging contaminants will continue to cause new and severe challenges to natural resources, ecosystems and human health. Importantly, the production and release of new chemicals in the environment will surpass the potential of available safety monitoring, risk assessment methods, preventative and remediation technologies [19]. In their review, Chauhan et al. (2018) showed the significant potential of nanomaterials for the degradation of pharmaceutically active compounds and other emerging contaminants from water matrices [53]. However, the authors underlined the lack of policies, regulatory guidelines and standard protocols about the use and safety issues of nanomaterials. In another recent interesting review, the simultaneous removal of pollutants from water using nanoparticles was presented [163]. Authors highlighted water multipollutant (pathogens, toxic organic and inorganic compounds) control by different nanomaterials (such as adsorbents, disinfectants and photocatalysts). The comprehension of the mechanisms that control the interactions between coexisting pollutants, and the application of these mechanisms for the development of novel nanocomposites composed of different nanoparticles with selectivity against different pollutants, are key elements for the treatment of multiple water pollutants. According to Gerbersdorf et al. (2015) risk assessment, management and modeling are key elements to tackle the emerging pollutants’ occurrence, fate and hazardous potential, and thus a multidisciplinary approach is required [18]. For the risk management, chemical analyses, human toxicology and biotoxicology, environmental impact assessments, economical and technical evaluations will be needed.

According to Kumar and Chowdhury (2020) the development of photocatalysts with enhanced properties and efficiency is expected to continue to be a challenging topic of research [166]. Some photocatalytic applications are commercially available and more will most probably be produced in the near future since the use of photocatalysts, and especially under visible light, constitutes a viable option for wastewater and water treatments. According to the authors, an out of the box thinking is required for new and economically feasible materials characterized by innovative structure and characteristics. Additionally, the process kinetics and scaling up issues have to be investigated in depth, and the strategy to use photocatalysis as a pretreatment step should be preferred because of the financial feasibility and better performance characteristics.

The following issues must be addressed in future studies regarding materials development: (i) long term efficiencies and fouling caused by direct contact, (ii) economic feasibility and sustainability, (iii) development of hybrid compounds with enhanced stability and low toxicity, (iv) optimized nanomaterial design with enhanced antibacterial activity, (v) large-scale synthesis/reproducibility and (vi) identification of potential adverse effects to human and the environment [145]. According to Qu et al., nanotechnology will support the development and application of easily operable, maintainable and replaceable point of use water treatment systems, especially in developing countries. Authors foresee that nanotechnology will reshape water supply systems to the direction of sustainability and smartness [37,162]. Efficient, eco-friendly and cost effective CNTs technologies for wastewater treatment may be developed in the near future applying new production and functionalization methods [9]. The improvement of electrocatalytic performances, the construction of heteroatom-doped carbon materials with the highest possible density of the most catalytically active groups and the use of various biologically derived materials as precursors for making heteroatom-doped carbon materials are among the most interesting research fields [173]. New developments will be boosted by the use of, e.g., diamond nanoparticles as support because they combine inertness with surface anchoring sites for metals or graphene oxides [174]. Tahir et al. have stressed out the limitations of graphene (i.e., it possesses many basal planes that are not catalytically active, thus inhibiting the charge transfer kinetics at the electrode–electrolyte interface), as well as the promising properties of graphene quantum dots and the significant potential to fabricate novel graphene-based nanocomposites with semiconductors, with a great variety of environmental applications [175]. Recently, Zhang et al. anticipated the future application of metal-free g–C_3_N_4_ nanocomposites (e.g., C60/g-C_3_N_4_, GO/g-C_3_N_4_ and S8/rGO/g-C_3_N_4_), especially for drinking water treatment, highlighting this technology as an emerging research hotspot [55]. Innovation in the synthesis of MOFs is one of the future developments and new ways may be opened for the production of NP/MOF catalysts or MOF-supported NP catalysts, which would possess characteristics and advantages of both heterogeneous and homogeneous catalysts [89]. Future work should focus on the MOF conversion and more attention should be paid on mechanistic investigations. The precise control of the final properties of MOF derivatives is still lacking, which will allow the customization of functional nanomaterials in practical applications [93].

Rizzo et al. (2019) reviewed on the consolidated compared to the new advanced treatment methods for the removal of emerging contaminants from wastewater. They commented that although advanced oxidation processes, and especially homogeneous and heterogeneous photocatalytic ones, have been found effective for the degradation of emerging contaminants, they have not yet found application at full scale [14].

Xiao et al. discussed future development trends in the area of photocatalytic AOPs including (i) the synthesis of reliable solar or visible light responsive photocatalysts, (ii) new reactor designs to resolve issues of mass transfer limitation for the immobilized photocatalyst, (iii) R&D of artificial light sources with longer lifetimes and (iv) mechanistic studies [42]. Extensive research investments are earmarked for the future on the use of nanomaterials for water purification, since they can efficiently remove biological, physical and chemical contaminants at the nanoscale. Development of hybrid compounds, toxicity issues, large scale synthesis and economic feasibility are among the parameters that have to be considered for the final practical application of nanomaterials in water sanitation [145]. Hossain et al. (2014) reviewed on the antimicrobial properties of nanomaterials, their applications and limitations in water treatment. They identified some nanomaterials with a significant antimicrobial potential (TiO_2_, ZVINP, MgO, nAg, ZnO, chitosan-based nanocomposites) [54]. In their review, Qu et al. (2013) stressed out the need to address the compatibility between the developed nanotechnologies and current water/wastewater treatment processes and infrastructure, since nanotechnology implementation has to be performed with minimal changes to existing facilities in the near term. According to the authors three categories were promising for full scale application that is nano-adsorbents, nanotechnology enabled membranes and nanophotocatalysts [162].

In the near future: (a) It is expected that more countries will regulate emerging pollutants in urban wastewater. Switzerland is the only country up to date. (b) Since ISO horizontal methods are released for the detection of different microbes in water matrices, and these analyses will become cost effective in the near future, it is also expected that more microbiological parameters will be also included in regulations, starting with virological ones. (c) Advanced oxidation processes, will find broader applications at the full scale level, providing economically feasible solutions for the abatement of emerging pollutants, following the significant rise of the last decade. Specifically solar driven processes will be adapted in areas with high solar irradiation levels. (d) Ideally, different and best suited technological solutions will be applied locally, based on the specific conditions. (e) Point-of-use or improved water treatment facilities will be based on innovative nanotechnologies and the application of nanomaterials. (f) A holistic strategy for the simultaneous abatement of multiple pollutants from water/wastewater matrices through the application of multifunctional nanotechnology will most probably prevail in future, when safety issues regarding the recovery of nanoparticles from treatment reactors will be achieved.

## Figures and Tables

**Table 1:** Selected publications on the development and application of carbon nanotubes, graphitic carbon nitride (CNT/g-C_3_N_4_) nanomaterials for advanced water treatment.

Nanomaterial	Name	Mechanism	Target	Light Irradiation	Efficiency	Reference
Ni−Ti Layered Double Hydroxide@Graphitic Carbon Nitride Nanosheet	g-C_3_N_4_@Ni−Ti LDH NCs	Photocatalytic/ Sonophotocatalytic	Antibiotics (amoxicillin-AMX)	Visible	99.5% AMX degradation within 75 min	[58]
Magnetic Fe_3_O_4_/multi-walled carbon nanotubes	Fe_3_O_4_/MWCNT	Catalytic wet peroxide oxidation	Diclofenac (DCF)		95% DCF removal after 3 h	[49]
Graphitic carbon nitride nanosheets	g-C_3_N_4_	Photocatalytic	Fluazaindolizine (FZDL)	Simulated sunlight	86% FZDL degradation after 18 h	[56]
Carbon quantum dots-modified porous g-C_3_N_4_	CQDs modified g-C_3_N_4_	Photocatalytic	Diclofenac (DCF)	Visible	15 times greater degradation than with pure g-C_3_N_4_	[59]
Multi wall carbon nanotubes (MWCNT)/TiO_2_ nano-composite	MWCNT/TiO_2_	Photocatalytic	Tetracyclin (TC)	UVC	TC removal after 100 min. 83% mineralization after 300 min. COD and TOCremoval of 84.9% and 82.3% for realpharmaceutical wastewater in 240 min.	[60]
Pd–Cu alloy NPs embedded in hollow octahedral N-doped porous carbon	Pd–Cu@HONPC	Catalytic oxidation	Hydrocarbons		Highest conversion of 89% for fluorene	[61]
Ti/RuO_2_–TiO_2_ electrode in the presence of multi-walled carbon nanotubes (MWCNTs)	Ti/RuO_2_–TiO_2_/MWCNTs	Electrocatalytic	Diclofenac (DCF)		99.8% DCF removal at 20 mA/cm^2^, current density	[62]

**Table 2:** Selected publications on graphene based composites for the treatment of emerging water micropollutants and pathogens.

Name/Nanomaterial	Method of Preparation	Target	Matrix	Light Irradiation	Efficiency	Reference
Fe(VI)-Fe_3_O_4_/GE system	co-precipitation method	ciprofloxacin	simulated water	visible light	98.5% of ciprofloxacin	[73]
silver decorated grapheme oxide (Ag/GO) composite	reduction process	*Escherichia coli*	aqueous solution	external light source (35 W Xenon lamp with emission spectra similar to the solar spectrum)	best sterilization under visible light for 60 min; bactericidal rate of 81.2–97.7%	[74]
SnO_2_-doped nanocomposites (SnO_2_ used as a dopant in sulphonated GO and CNT)	hydrothermal method	*Escherichia coli* and *Pseudomonas graminis*, isolated from wastewater treatment plant	TSB petri plates/disc diffusion method	visible light	photocatalytic SnO_2_-doped nanocomposites induced 50% antibacterial activity against *E. coli* and *P. graminis*	[75]
Ag NPs/GA composite homogenously loaded on graphene aerogel (GA)	hydrothermal method	*Escherichia coli* and 4-nitrophenol	deionized water	none	bactericidal performance for 8–lg of *E. coli* cells with 100% inactivation rate and catalytic activity for 4-NP with 96.6% degradation rate	[76]
nano zinc oxide incorporated graphene oxide/nanocellulose (ZnO-GO/NC) nano composite	GO by modified Hummers and Offman’s method; Nano cellulose from cellulose by sulphuric acid hydrolysis	ciprofloxacin	aqueous solution (and superficial water samples)	visible light	maximum degradation efficiency of 98% for ciprofloxacin	[68]
nickel doped CdS nanoparticles anchored on graphene nanosheets (G-NiCdS)	microwave-furnace assisted method	cephalexin and sulfamethoxazole	aqueous solution	visible light	cephalexin almost eliminated within 180 min (95%); sulfamethoxazole removed to (95%) within 240 min by G-NiCdS	[77]

**Table 3:** Selected publications on metal organic frameworks (MOFs)-based nanomaterials for the treatment of emerging water micropollutants and pathogens.

Name/Nanomaterial	Method of Preparation	Target	Light Irradiation	Efficiency	Reference
yolk-shell Co3O4@MOFs nanoreactor	one-pot solvothermal method	4-chlorophenol	none	almost 100% within 60 min (in the presence of peroxymonosulfate)	[100]
UiO-66@AgI	solvothermal method/in situ growth method	sulfamethoxazole	visible light	99.6% of the sulfamethoxazole (5 ppm) in 20 min	[101]
Pd@MIL-100(Fe) nanocomposite	alcohol reduction	ibuprofen, theophylline and bisphenol A	visible light	ibuprofen (69.2), theophylline (45.2) and bisphenol A (20.5)	[102]
core–shell In_2_S_3_@MIL-125(Ti) (MLS) photocatalytic adsorbent	solvothermal method	tetracycline	visible light	photodegradation efficiency of 63.3%	[103]
Fe-based metal organic framework (MOF) (viz. MIL-100(Fe))	hydrothermalmethod	sulfamethoxazole, and additionally carbamazepine, cephalexin, ciprofloxacin, tetracycline (in real wastewater matrices)	visible light	98.9% for sulfamethoxazole (>90% in different wastewater conditions)	[104]
metal organic framework MIL-100(Fe) with FeII/FeIII mixed-valence coordinatively unsaturated iron center (CUS-MIL-100(Fe))	hydrothermal method	sulfamethazine	none	100% within 3 h	[105]
MIL-101(Fe)@TiO2	solvothermal method	tetracycline	solar light	92.76% and 93.8% in 10 min	[106]
polylactic acid (PLA) fibers containing Co-SIM-1, a cobalt-based substituted imidazolate	electrospinning	*Pseudomonas putida* and *Staphylococcus aureus*	none	higher sensitivity of *S. aureus* to cobalt-containing fibers, with a reduction in colony forming units of up to 60% with respect to PLA mats	[107]
Four mixed Ti-Zr-MOFs (TiZr15, TiZr30, TiZr60, TiZr80) by partial substitution of Ti by Zr atoms in the crystalline structure of NH2-MIL-125(Ti) MOF	solvothermal method	acetaminophen	solar light	crystalline TiZr15 yielded the highest activity (100% of acetaminophen after 90 min)	[108]
[Zn2(fum)2(bpy)] and [Zn4O(bdc)3] (fum = fumaric acid; bpy = 21 4,4-bipyridine; bdc = benzene-1,4-dicarboxylate) metal–organic frameworks (MOFs)	solid state approach	amodiaquine drug	none	maximum adsorption capacities for amodiaquine of 0.478 and 47.62 mg/g on the [Zn2(fum)2(bpy)] and [Zn4O(bdc)3] MOFs, respectively	[109]

## Abbreviations

A/TNS	anatase/titanate nanosheets
AOPs	advanced oxidation processes
BPA	bisphenol A
CAS	copper aluminosilicate
CB	conduction band
CB	cuttlefish bone
CNT	carbon nanotubes
CNTs	carbon nanotubes
COD	chemical oxygen demand
CQDs	carbon quantum dots
CTAB	cetyltrimethylammonium bromide
DNA	deoxyribonucleic acid
DOX	doxorubicin
EG	ethylene glycol
G/A/TNS	graphene modified anatase/titanate nanosheets
GA	graphene aerogel
g-C_3_N_4_	graphitic carbon nitride
GO	graphene oxide
GO/TNA	graphene oxide deposited TiO_2_ nanotubes array
LED	light-emitting diode
MOFs	metal organic frameworks
MWCNT	multi wall carbon nanotubes
NCMC	nanocarbon–metal composition
NPs	nanoparticles
nZVI	nano zero-valent iron
PAHs	polycyclic aromatic hydrocarbons
PEG	polyethylene glycol
PET	polyethylene terephthalate
PLA	polylactic acid
PPCP	personal care product
PVA	polyvinyl alcohol
PVP	polyvinylpyrrolidone
rGO	reduced graphene oxide
SiO_2_@Fe_3_O_4_	core–shell magnetic silica
TOC	total organic carbon
TPC	total phenolic content
UV	ultraviolet
VB	valence band
